# Wanted: A New Model for Glucocorticoid Receptor Transactivation and Transrepression

**DOI:** 10.1371/journal.pbio.1001814

**Published:** 2014-03-18

**Authors:** Caitlin Sedwick

**Affiliations:** Freelance Science Writer, San Diego, California, United States of America

Glucocorticoid receptor (GR) is a transcription factor that is almost ubiquitous in vertebrate cells. As one of the cellular receptors for steroid hormones of the glucocorticoid (GC) family, which are well known for their anti-inflammatory properties in clinical applications, the regulation of GR activity has been the focus of intense study.

Upon binding GC, GR translocates from the cytoplasm to the nucleus, where it may either bind to and activate transcription from gene promoters (transactivation) or interact with other transcription factors to alter their function (transrepression). These two modes have very different effects, and GR transactivation is linked to many of the negative side effects of GCs that are used therapeutically. Several groups are therefore searching for GR ligands that can separate GR's transactivation and transrepression activities. However, the search is hampered because the mechanisms of GR transactivation and transrepression are poorly understood. In this month's *PLOS Biology*, Diego M. Presman, Adali Pecci, Gordon L. Hager, and colleagues report on their efforts to clarify GR's different modes of activity.

GR can form a dimer with itself, and one popular model holds that the GR dimer transactivates genes, whereas monomeric GR participates in transrepression. This suggestion was based on earlier work with a mutant version of GR called GRdim, which was reported to participate in transrepression, but not transactivation, and was thought to be deficient in dimerization. However, newer research has challenged these findings, so Presman et al. sought to determine whether GR dimerization is required for transactivation.

**Figure 1:**
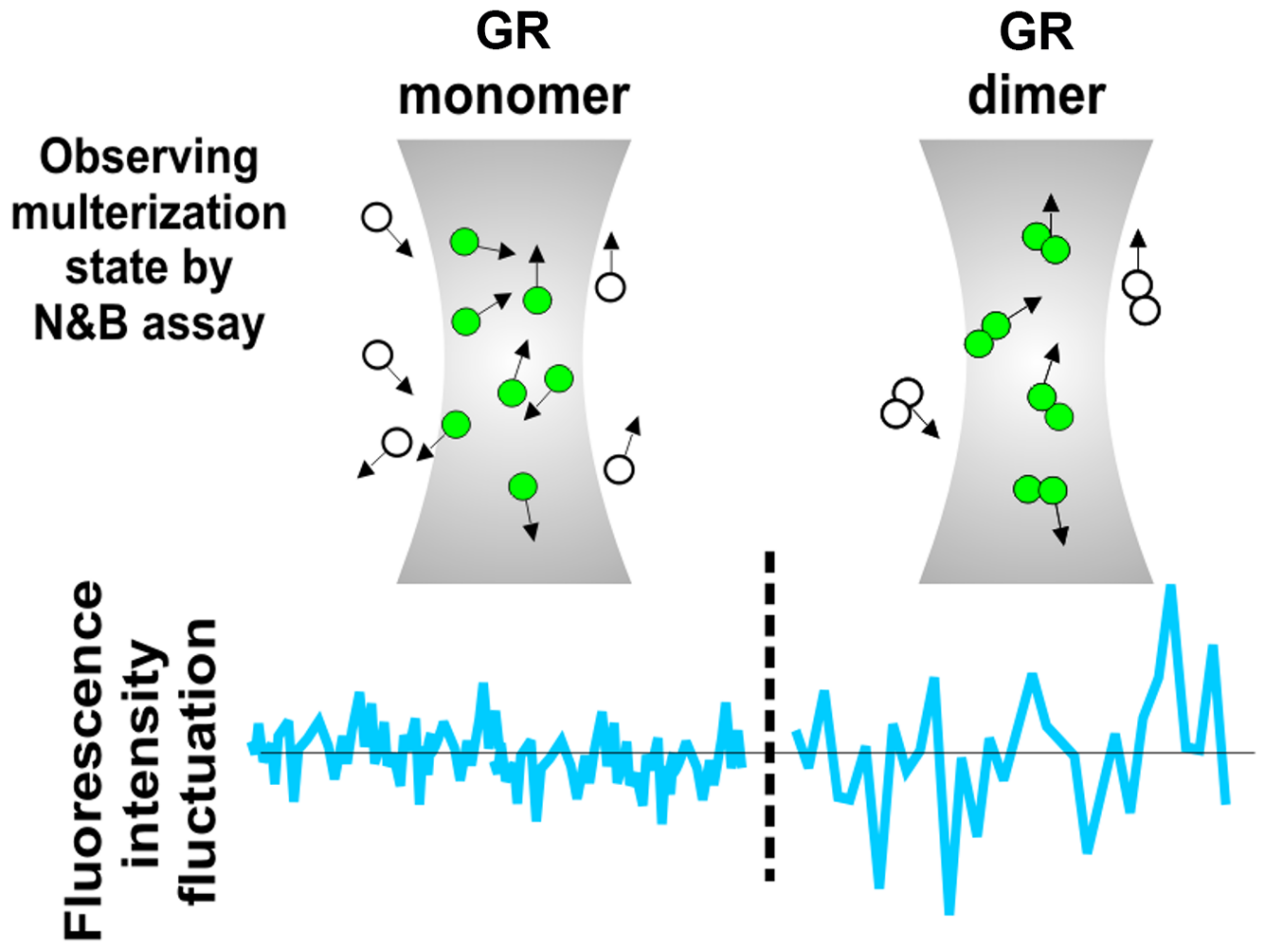
The monomer–dimer equilibrium for interacting molecules can be observed in living cells with fluorescence intensity fluctuation spectroscopy. Using the Number and Brightness (N&B) version of this methodology, the dimerization state for glucocorticoid receptor (GR) was observed for GR variants with mutations in domains that participate in contact between the receptor subunits.

Presman et al. used a microscopy technique called the Number and Brightness (N&B) assay, which can detect whether a fluorescently tagged protein has dimerized by measuring pixel-intensity fluctuations in captured images of cultured cells (Figure 1). As expected, in untreated mouse or hamster cells expressing Green Fluorescent Protein-tagged GR, GR was mostly found in the cytoplasm. Further, treatment with a GR ligand caused GR to translocate to the nucleus, where N&B detected its dimerization. However, N&B also detected dimerization of GRdim following ligand exposure, indicating that this mutant can dimerize after all.

Besides its purported inability to dimerize, GRdim was thought to be incapable of transactivation because it could not bind to DNA. Presman et al. tested this in vivo using a cell line that contains an artificial stretch of DNA with a tandem array of GR-binding sites and found that, like wild-type GR, GRdim could bind to this array. In addition, when the authors examined GR occupancy of specific DNA-binding sites using chromatin immunoprecipitation, they found that GRdim simply occupied with less efficiency the sites to which wild-type GR could bind. Therefore, impaired transactivation by GRdim is explained neither by an inability to dimerize nor by an inability to bind to DNA.

Presman et al. next examined GR's transrepression efficiency. Previously, it was thought that constitutively monomeric GRdim could preferentially bind to and modulate activity of other transcription factors, such as the NF-κB subunit, p65. However, cross-correlation image analysis suggested that even dimerized GRdim or dimerized wild-type receptor could interact with p65. While these experiments could not rule out the possibility that monomeric GR interacts with p65, they underlined the fact that GRdim cannot be used to test adequately how GR dimerization status affects the receptor's cellular activities. This result prompted the authors' attempt to create a GR mutant that was truly incapable of dimerization.

Structural studies have identified two potential surfaces where dimerized GR proteins may contact each other. One of these, in the protein's DNA-binding domain, is disrupted in the GRdim mutant. Meanwhile, the other is located in a different part of the molecule, the ligand-binding domain. A synthetic GR ligand known to disrupt the ligand-binding domain did not affect dimerization of wild-type GR. However, it did block dimerization of the GRdim mutant, indicating that both the ligand-binding and DNA-binding domains of GR contribute to dimerization. In agreement with this idea, Presman et al. found that a GR protein containing disruptive mutations in both domains exhibited severely compromised dimerization. Accordingly, they dubbed this dual mutant GRmon (“mon” is short for “monomeric”).

Interestingly, like GRdim, GRmon showed impaired transactivation activity yet was still capable of transrepression, although GRmon was much less efficient than GRdim at binding to DNA. Altogether, their data led Presman et al. to conclude that, while GR dimerization does affect its DNA-binding efficiency, the protein's dimerization status does not correlate with its transcriptional activity. Therefore scientists must find a different explanation for the separability of GR transactivation and transrepression.

**Presman DM, Ogara MF, Stortz M, Alvarez LD, Pooley JR, et al. (2014) Live Cell Imaging Unveils Multiple Domain Requirements for** ***In Vivo*** **Dimerization of the Glucocorticoid Receptor.** doi:10.1371/journal.pbio.1001813

